# Learning from sotorasib: risk of bias in confirmatory clinical studies of accelerated approved drugs and resolution strategies

**DOI:** 10.1186/s12916-024-03414-y

**Published:** 2024-05-20

**Authors:** Ning Jiang, Yanjie Han, Shuhang Wang, Ning Li

**Affiliations:** Clinical Trial Center, National Cancer Center/National Clinical Research Center for Cancer/Cancer Hospital, Chinese Academy of Medical Sciences, Peking Union Medical College, 17 South Panjiayuan Lane, Chaoyang District, Beijing, 100021 People’s Republic of China

**Keywords:** Systemic bias, Clinical trial, Accelerated approved drugs, Resolution strategy, Sotorasib

## Background

On May 28, 2021, the FDA granted accelerated approval to sotorasib, the first targeted therapy for the treatment of locally advanced or metastatic non-small cell lung cancer patients with KRAS G12C mutations who had received at least one prior systemic therapy [1]. This approval was based on the single-arm CodeBreaK 100 study (NCT03600883) [2]. As part of the postmarketing requirement (PMR) to validate the clinical benefit, a phase III, open-label, randomized CodeBreaK 200 study (NCT04303780) was initiated. As a result, the primary endpoint, progression-free survival (PFS) assessed by blinded independent central review (BICR), was achieved [3].

The FDA’s accelerated approval pathway is designed to expedite the availability of therapies that treat serious conditions and fill unmet medical needs, usually based on a surrogate endpoint. Though sotorasib received accelerated approval and achieved the primary endpoint in phase III study, limitations of CodeBreaK 200 were also highlighted by other authors. Olivier et al. pointed out that the control arm did not meet the best standard of care and underwent a protocol change that reduced the sample size and permitted crossover, impeding the assessment of OS [4]. Additionally, an imbalance in censoring rates raised concerns over the reliability of the PFS estimates [4].

On October 5, 2023, at the Oncologic Drugs Advisory Committee Meeting (ODAC), the FDA questioned the confirmatory clinical trial, CodeBreaK 200 study [5]. Primary issues encompassed the potential overestimation of PFS benefits due to imbalanced early dropout, investigator imaging assessments favoring sotorasib, and the influence of investigator patient management on BICR-assessed PFS [5]. The committee’s vote centered on the following question: can the primary endpoint, PFS per BICR, be reliably interpreted in CodeBreaK 200? Ultimately, 10 out of 12 academic committee members concurred that PFS benefit of CodeBreaK 200 lacked reliable interpretability. In the wake of these deliberations, FDA mandated a new PMR for an additional confirmatory study to support full approval, which needs to be completed no later than February 2028 [6].

The discussion highlights the crucial issue of potential bias in confirmatory clinical trials for drugs granted accelerated approval. In this commentary, we will summarize the bias in CodeBreaK 200 and discuss the potential strategies to mitigate bias in confirmatory trials of drugs granted accelerated approval.

## Main text

### Randomization limitation

At the ODAC meeting, a possible source of bias identified by the FDA was the disproportionate early dropout rate, with greater dropout in the docetaxel group (13% vs. 1%), potentially compromising the study’s randomization integrity. In the context of randomized phase III studies of drugs that have received accelerated approval, which are commonly employed in PMR as mandated by the FDA, both participants and investigators are susceptible to information bias. This bias stems from the presumption that the experimental drug outperforms the control. Consequently, control group participants are prone to premature dropout and a propensity to report enhanced outcomes for the experimental drug. Investigators may prematurely identify progression in the control group and delay the determination of progression in the experimental group.

To mitigate bias related to randomization, the FDA suggested patient and investigator education and suggested allowance of crossover. Another quintessential approach is the double-blind design, employed in numerous phase III trials, including KEYNOTE-091 and NCT00094653, in which intravenous placebo controls were used [7, 8] (concluded in Table 1). Nonetheless, this methodology can significantly complicate trial execution, given the disparities in adverse reaction spectra, administration procedures, and pre-treatment protocols between therapies. For therapies with preexisting accelerated approval, facilitating recruitment and upholding ethical standards often necessitates permitting control group patients to transition to the experimental cohort post-progression. In such instances, maintaining a blind design proves impractical. A viable alternative involves authorizing patient unblinding post-progression, allowing for a subsequent crossover to the experimental treatment regimen.

**Table 1:** Bias risk in confirmatory clinical studies of accelerated approved drugs and resolution strategies

	**Resolution strategies**				
	Double-blind design	Allowance of crossover	Real-time BICR	OS endpoint	pRCT assist evaluation
**Risk**					
**Information bias from participants**					
Control group participants tend to drop out early	√	√	-	-	-
Bias in patient reported endpoints	√	-	-	√	-
**Information bias from researchers**					
Early determination of progression in control group	√	-	√	√	-
Delayed determination of progression in experimental group	√	-	√	√	-
**Bias arising from imaging assessments**	√	-	√	√	-
**External validity issues**					
Deviation from real-world condition	-	-	-	-	√

√ indicates potential useful strategies to mitigate bias

*BICR* blinded independent central review, *OS* overall survival, *RWS* real-world study, *pRCT* pragmatic randomized controlled trials

### Outcome limitation

In the CodeBreaK 200 study, PFS evaluated by BICR served as the primary endpoint. Nevertheless, the FDA review team highlighted concerns about the reliability of the results, given its scale compared to the frequency of imaging assessments (5 weeks versus 6 weeks) and the discordance between investigators and BICR, which indicated the existence of bias.

The judicious choice of primary endpoints can mitigate bias. While both PFS and OS are sanctioned as endpoints for standard approval, PFS, being a composite endpoint of imaging assessment and overall survival, may introduce bias if there is information bias in disease progression assessment by imaging evaluators. Moreover, as a surrogate endpoint, PFS does not always robustly predict OS benefits. In NSCLC, the correlation between PFS and OS is not well established, especially in studies with crossover design [9]. Conversely, OS, a clinical endpoint, remains impervious to information bias during evaluation. Nonetheless, OS is heavily swayed by subsequent-line treatments. For drugs with prior accelerated approval, crossover after disease progression may diminish OS discrepancies, complicating the attainment of a favorable outcome.

Employing blinded independent central review (BICR) is crucial for countering inherent biases in investigator assessments, especially in open-label trials of conditionally approved drugs, where investigators might perceive these treatments as efficacious. In CodeBreaK 200, BICR was designated for disease response evaluation, yet an independent central confirmation of progression (COP) was instituted as well. The COP was primarily introduced for the timely crossover decision but has led to a series of challenges in imaging evaluation, including the premature crossover prior to BICR-assessed progression and the BICR re-read [5]. Radiologist discrepancies, particularly in borderline cases, are common. Therefore, early crossover might be inevitable if the crossover decision and endpoint assessment involve different radiologist teams. To address this issue, we recommend exclusively relying on real-time BICR for the crossover decision, which could address both promptness and early crossover censoring.

### External validity

Controlling crossover in RCTs for drugs granted accelerated approval could be nearly impractical, as patients can readily access the drug through alternative means. Drugs granted accelerated approval often amass substantial real-world clinical data, yet conventional RCTs fail to leverage this information effectively. Furthermore, the rigid inclusion criteria of RCTs hinder an accurate reflection of these drugs’ performance in real-world applications. Hence, we advocate concurrently exploring alternative research modalities, such as real-world studies (RWS), to expedite full approval.

Currently, most of the RWS are observational studies, collecting data mainly from the medical records. However, such design inherited the same limitations of traditional observational studies, including the bias introduced by non-randomization. Pragmatic randomized controlled trials (pRCTs) with the randomization process could be a more suitable design. pRCTs can represent real-world conditions more closely and can yield extensive safety data, potentially boasting superior external validity and preserving the advantages of randomization. Within pRCTs, issues like open-label design and non-adherence, prevalent in actual clinical environments and potentially affecting treatment efficacy, do not pose substantial challenges. While biases remain possible in pRCTs, a meticulously structured pRCT with a sufficiently large cohort can provide reliable evidence. Simultaneously considering the results of both pRCTs and well-designed RCTs may lead to more robust and dependable conclusions.

Overall, bias in the confirmational clinical trials of drugs granted accelerated approval may originate from multiple sources. Risk of bias in randomized trials could be evaluated by tools like Risk of Bias 2 (RoB 2), which contained five domains assessing bias related to the randomization process, deviations from intended interventions, missing outcome data, and so on [10]. Besides, when designing a postmarketing confirmational trial, strategies to mitigate bias are necessary. Feasible strategies may include double-blind design, choice of objective endpoints, and reliable methods for assessment (Table 1). Furthermore, adequate sample size to mitigate the influence of dropout and crossover might be needed. Among these, the double-blind design might be one of the most crucial strategies, potentially able to minimize all the biases arise from the participants, the researchers, and the image assessment processes.

## Conclusions

In summary, we posit that drugs benefiting from accelerated approval encounter a pronounced risk of bias in subsequent confirmatory clinical trials. To circumvent disruptions in clinical effect observations, potential biases must be thoroughly anticipated at the design phase, with strategic actions implemented to minimize bias. Rigorous adherence to the protocol throughout the study’s duration is imperative, complemented by measures ensuring patient compliance.

## Data Availability

Not applicable.
